# *In vivo* Microscale Measurements of Light and Photosynthesis during Coral Bleaching: Evidence for the Optical Feedback Loop?

**DOI:** 10.3389/fmicb.2017.00059

**Published:** 2017-01-24

**Authors:** Daniel Wangpraseurt, Jacob B. Holm, Anthony W. D. Larkum, Mathieu Pernice, Peter J. Ralph, David J. Suggett, Michael Kühl

**Affiliations:** ^1^Marine Biological Section, Department of Biology, University of CopenhagenHelsingør, Denmark; ^2^Climate Change Cluster, Department of Environmental Sciences, University of Sydney, SydneyNSW, Australia

**Keywords:** coral bleaching, *Symbiodinium*, photosynthesis, light scattering, coral optics, ecophysiology

## Abstract

Climate change-related coral bleaching, i.e., the visible loss of zooxanthellae from the coral host, is increasing in frequency and extent and presents a major threat to coral reefs globally. Coral bleaching has been proposed to involve accelerating light stress of their microalgal endosymbionts via a positive feedback loop of photodamage, symbiont expulsion and excess *in vivo* light exposure. To test this hypothesis, we used light and O_2_ microsensors to characterize *in vivo* light exposure and photosynthesis of *Symbiodinium* during a thermal stress experiment. We created tissue areas with different densities of *Symbiodinium* cells in order to understand the optical properties and light microenvironment of corals during bleaching. Our results showed that in bleached *Pocillopora damicornis* corals, *Symbiodinium* light exposure was up to fivefold enhanced relative to healthy corals, and the relationship between symbiont loss and light enhancement was well-described by a power-law function. Cell-specific rates of *Symbiodinium* gross photosynthesis and light respiration were enhanced in bleached *P. damicornis* compared to healthy corals, while areal rates of net photosynthesis decreased. *Symbiodinium* light exposure in *Favites* sp. revealed the presence of low light microniches in bleached coral tissues, suggesting that light scattering in thick coral tissues can enable photoprotection of cryptic symbionts. Our study provides evidence for the acceleration of *in vivo* light exposure during coral bleaching but this optical feedback mechanism differs between coral hosts. Enhanced photosynthesis in relation to accelerating light exposure shows that coral microscale optics exerts a key role on coral photophysiology and the subsequent degree of radiative stress during coral bleaching.

## Introduction

Solar radiation governs coral photophysiology and ultimately drives the productivity and growth of coral reefs (Falkowski et al., 1990). Light stimulates photosynthesis of coral microalgal endosymbionts (*Symbiodinium* spp.), generating O_2_ and carbohydrates that are exported to the host fueling coral animal metabolism (Muscatine et al., 1981). However, excess light enhances *Symbiodinium* photodamage (Warner et al., 1999; Takahashi et al., 2004) and, in combination with anomalous seawater temperatures, can induce the breakdown of the coral-algal symbiosis known as coral bleaching (Brown, 1997; Hoegh-Guldberg, 1999). Coral bleaching events are regarded as a major threat to the future of coral reefs (Ainsworth et al., 2016) and hence the physiological mechanisms triggering coral bleaching have been a major research focus for decades (Weis, 2008). Coral bleaching susceptibility is affected by a combination of factors that act on different spatial and temporal scales, including coral thermal history (Brown et al., 2002; Hughes et al., 2003), *Symbiodinium* genotype (Sampayo et al., 2008), as well as biochemical pathways and tissue properties of the coral host species (Baird et al., 2009). At the cellular scale, coral bleaching involves enhanced thermal and radiative exposure of *Symbiodinium* cells, resulting in photodamage and the subsequent generation of reactive oxygen species (ROS) that induce the breakdown of the symbiosis (Lesser, 1996; Hoegh-Guldberg, 1999; Weis, 2008). The *in vivo* light and temperature exposure of *Symbiodinium* within the host tissue ultimately controls whether *Symbiodinium* undergoes photodamage, and it is thus important to resolve the optical and thermal microenvironment of coral hosts (Enriquez et al., 2005; Jimenez et al., 2008; Wangpraseurt et al., 2012; Swain et al., 2016).

Application of light microsensors has shown that the *in vivo* light exposure within coral tissues can be enhanced over the incident downwelling irradiance (Kühl et al., 1995; Wangpraseurt et al., 2012, 2014a,b; Brodersen et al., 2014). Such irradiance enhancement is modulated by the unique optical properties of coral tissue and skeleton (Enriquez et al., 2005; Teran et al., 2010; Kahng et al., 2012; Marcelino et al., 2013; Wangpraseurt et al., 2016a) and can improve photosynthesis under low light conditions (Brodersen et al., 2014; Wangpraseurt et al., 2014a) or lead to light stress under high irradiance (Marcelino et al., 2013; Swain et al., 2016). The loss of *Symbiodinium* spp. cells from corals under environmental stress has been hypothesized to further increase irradiance exposure *in hospite* due to decreased shading by photopigments and increased backscattered light from the coral skeleton (Enriquez et al., 2005; Teran et al., 2010; Swain et al., 2016). According to this so-called optical feedback hypothesis (Enriquez et al., 2005; Swain et al., 2016), skeleton backscattering can further stimulate symbiont loss inducing an accelerating cycle, where symbiont loss promotes light enhancement and vice versa. Several studies have speculated on the relevance of such a feedback loop exacerbating coral bleaching, arguing that the light microenvironment during a stress event could serve as a key factor in determining the severity of a bleaching event (Hoegh-Guldberg, 1999; Franklin et al., 2006; Hoogenboom et al., 2006; Abrego et al., 2008; Weis, 2008; Baird et al., 2009). However, experimental proof of such a mechanism has been lacking.

Here, we provide the first direct measurements of the *in vivo* light environment of *Symbiodinium* during coral bleaching. We performed a thermal stress experiment and monitored changes in the *in vivo* light environment using light microsensors in two coral species with contrasting optical properties in concert with O_2_ microsensor-based measurements of gross photosynthesis, net photosynthesis and light respiration. We provide evidence for the acceleration of *in vivo* light exposure upon coral bleaching and show that such light enhancement differs between coral hosts. This highlights the importance of skeleton and tissue optics for coral photophysiology and stress responses.

## Materials and Methods

### Coral Species

Light microenvironments were investigated in two shallow-water corals: the branching coral *Pocillopora damicornis*, and the massive coral *Favites* sp. The coral *P. damicornis* is generally a bleaching-susceptible species (Loya et al., 2001; Swain et al., 2016) with thin tissues (maximum thickness ∼150 and 300 μm for coenosarc and polyp tissues, respectively) and low tissue light attenuation (Szabó et al., 2014). *P. damicornis* from Heron Island is known to harbor different *Symbiodinium* spp. including symbiont type C42ab and C33a (Tonk et al., 2013). *Favites* sp. is a more bleaching-resistant species (Swain et al., 2016) with a polyp tissue thickness of ∼2 mm (Wangpraseurt et al., 2014a) and is known to associate with symbionts of type C1, C21, and C33 (Tonk et al., 2013). Corals were collected at low tide from shallow waters (<2 m depth) on the reef flat off Heron Island, Great Barrier Reef, Australia (152°06′ E, 20°29′ S) in August–September 2014. On a cloudless day during mid-day sun, the incident downwelling photon irradiance integrated over 400–700 nm, i.e., PAR, reached *in situ* values at the level of the sampled colonies of E_d_(PAR) > 2000 μmol photons m^-2^ s^-1^ (Wangpraseurt et al., 2014b). Corals were selected from the same small sample area in order to ensure that they were adapted to a comparable light and flow regime. Coral fragments from at least 10 different daughter colonies of *P. damicornis* and *Favites* sp. were collected and sub-fragmented (*P. damicornis* ca. 3–10 cm in length and *Favites* ca. 3 cm in diameter).

### Bleaching Experiment

Collected corals were allowed to recover from potential translocation stress for a minimum of 1 week in a shaded outdoor aquarium at Heron Island Research Station before transfer to the experimental incubation set-up consisting of an outdoor aquarium tank (300 L) that was exposed to natural sunlight (13 h of daylight) and continuously supplied with seawater from the reef flat at an exchange rate of about 0.2 L s^-1^. The set-up mimicked conditions on the Heron Island reef flat for a water depth of 20–30 cm with high solar irradiance exposure. A water heater connected to a thermistor (accuracy: ±1.5°C, Sensor type: KTY81-121, Sentien Electronics, Australia) was placed in one corner of the tank and two submersible water pumps directed the water flow (3–5 cm s^-1^) toward the heater, while four additional water pumps recirculated the water in the tank at a comparable flow speed in order to avoid the build-up of temperature gradients within the tank. E_d_(PAR) during noon was ∼2000 μmol photons m^-2^ s^-1^ as measured at the water surface with a planar quantum irradiance sensor connected to a light meter (LI-190 and LI-1400, Li-Cor, Lincoln, NE, USA). Temperature was monitored using data loggers (Onset, USA) randomly distributed within the tank and logging at a time interval of 1 min. Coral fragments were randomly distributed within the tank. In order to induce coral bleaching, we followed the protocol of Middlebrook et al. (2010), whereby the water temperature was step-wise increased by about 1°C per day. In total the experiment ran for 13 days during September 2014, and included 3 days of baseline measurements at ambient water temperature (22°C) followed by 10 days ramping of temperature from 22 to 33°C.

Often experimental measurements on coral bleaching use a balanced experimental design where temperature treated corals are assessed on par with healthy corals during the course of the experiment. In the microenvironmental approach used here, the total number of samples was limited by the experimental challenges involving intra-tissue microsensor measurements with a high chance of sensor breakage. As our main aim was to assess the optical mechanisms underlying coral bleaching, we prioritized measuring coral tissues with reduced algal cell densities over a larger number of control measurements on healthy corals. It is important to note that our experimental design does thus not allow for excluding potential experimental treatment effects (e.g., coral disease) on the physiology of the algae. However, all corals were visually expected for tissue necrosis and sloughing, and corals appeared visually free of disease. During the temperature ramping experiment, visual bleaching of *Favites* sp. occurred much slower than in *P. damicornis* and we thus accelerated the bleaching process for *Favites* sp. through a combined temperature and light stress treatment (see Supplementary Methods). Our approach did not allow us to compare differences in photophysiology or the rate of symbiont loss between *P. damicornis* and *Favites* sp.

### Optical Measurements

Spectral scalar irradiance E_0_(λ) and reflectance *R*_H_(λ) measurements were performed in a flow chamber that was flushed at a flow rate of 0.5–1 cm s^-1^ with seawater at the same temperature as in the experimental bleaching treatment tank. Fiber-optic scalar irradiance microprobes with a spherical tip diameter of 80 μm (Rickelt et al., 2016) were used to measure the spectral light microenvironment on and within coral coenosarc and polyp tissues as described previously (Wangpraseurt et al., 2012; see Supplementary Methods for details).

Spectral reflectance measurements on intact corals (*R*_H_) were performed with a flat-cut fiber-optic reflectance probe (2 mm diameter, Ocean Optics, USA) positioned at a 45° angle relative to the coral surface at a distance of 1 cm from the sample, using a similar measuring configuration as in Rodriguez-Roman et al. (2006). Vertically incident light [E_d_(PAR) = 400 μmol photons m^-2^ s^-1^] was provided by a fiber-optic tungsten halogen-lamp equipped with a collimating lens (Schott KL-2500, Germany). The coral reflected light measurements were normalized to the reflected light measured under identical optical configuration from a 99% white reflectance standard (Spectralon, Labsphere, USA). Scalar irradiance and reflectance spectra were also measured on bare skeletons (see Supplementary Figure S1).

### Photosynthesis and Respiration Measurements

Clark-type O_2_ microsensors (tip size of 25 μm, a 90% response time of <0.5 s and a stirring sensitivity of ∼1%; Unisense A/S, Aarhus, Denmark) were used to measure O_2_ production and consumption of *P. damicornis* as described previously (Wangpraseurt et al., 2012; Brodersen et al., 2014). Sensor readings were linearly calibrated from measurements in air saturated water and anoxic water (flushed with N_2_). Percent air saturation in seawater at experimental temperature and salinity was transformed to O_2_ concentration (μmol O_2_ L^-1^) using gas tables (Ramsing and Gundersen, Unisense, Denmark; www.unisense.com). All O_2_ microsensor measurements were performed in the same configuration as the scalar irradiance microsensor measurements but with the O_2_ microsensors approaching the coral surface at an angle of ∼10° relative to the vertically incident light. Measurements of gross photosynthesis at the coral surface were performed using the light–dark shift technique (see Revsbech and Jorgensen, 1983 for detailed description) followed by measurements of steady-state O_2_ concentration profiles from the coral tissue surface through the diffusive boundary layer (DBL) and into the mixed turbulent water phase above (Brodersen et al., 2014) under an incident photon irradiance of E_d_(PAR) = 400 μmol photons m^-2^ s^-1^. Steady-state O_2_ conditions were usually achieved within 5–10 min at a constant light level and each microsensor profile took on average about 5 min. We chose E_d_ = 400 μmol photons m^-2^ s^-1^ as a irradiance level close to the photosynthetic maximum based on previous microsensor measurements under comparable conditions (Ulstrup et al., 2006) and preliminary results from variable chlorophyll fluorimetry-based measurement of relative photosynthetic electron transport rates vs. irradiance (data not shown). The coral was then placed in darkness for >15 min before O_2_ profiles measured the dark respiration of the coral holobiont (Schrameyer et al., 2014). Additional measurements of the dark-adapted maximum quantum yield of PSII were done with a commercial variable chlorophyll fluorescence imaging system (Imaging-PAM, Walz GmbH, Germany; see supporting information and Supplementary Figure S2).

### *Symbiodinium* Density and Pigment Dynamics

*Symbiodinium* cell density was determined for small tissue areas of *Favites* sp. using a microsampling technique (Kemp et al., 2008), where individual polyps were sampled by careful separation of the coral tissue from the underlying skeleton using a syringe and needle (see supporting information and Supplementary Figure S3). This technique was not used for *P. damicornis* since the thin tissue of this coral was firmly attached to the skeleton, making it difficult to completely remove the tissue with a syringe. Instead small branch tips (maximum of 0.5–0.8 cm in length) were collected, crushed and centrifuged to separate host tissue, skeleton and *Symbiodinium* (see supporting information). For both coral species, *Symbiodinium* density was then estimated using a haemocytometer. Additional tissue samples were collected (as described above) for HPLC (high performance liquid chromatography) analysis using standard protocols (see supporting information).

### Data Analysis

Measured raw spectra were integrated between 400 and 700 nm (PAR) using the mathematical integration function of Origin Pro 9.1 (Origin, USA). The *in vivo* light enhancement relative to the incident downwelling irradiance was calculated as E_0_(PAR)/E_d_(PAR). Data was averaged for measurements performed on day 1 (‘healthy,’ water temperature 22°C) and day 10 (‘bleached,’ water temperature 33°C). We also expressed the enhancement of spectral scalar irradiance, E_0_(λ), of bleached corals relative to healthy corals as E_0_(λ)_bleached_/E_0_(λ)_healthy_. Areal rates of gross photosynthesis (PG; nmol O_2_ cm^-2^ s^-1^) were calculated by depth integration of the volumetric rates assuming constant rates of photosynthesis over the entire tissue. Tissue thickness of *P. damicornis* was ca. 300 μm for polyp tissues and ca. 150 μm for coenosarc tissues as determined with scalar irradiance microsensors (data not shown, see Wangpraseurt et al., 2014a). Net photosynthesis (PN) and dark respiration (RD) rates were calculated using Fick’s first law of diffusion (see Chan et al., 2016 for details). Light respiration of the coral holobiont (LR) was then calculated as the difference between gross and net photosynthesis (Schrameyer et al., 2014). In order to compare *Symbiodinium* dependent gross photosynthetic rates between healthy and bleached corals, we normalized our areal rates to cell density. Values of O_2_ production in nmol O_2_ cm^-2^ s^-1^ was divided by the average measured cell density (in cells per cm^-2^) to express gross photosynthesis in fmol O_2_ cell^-1^ s^-1^.

### Statistical Analysis

Regression analyses were used to test for the relationship between *Symbiodinium* cell density and *in vivo* coral tissue scalar irradiance. A non-linear curve fitting procedure was applied to test the relationship between the *in vivo* E_0_ (expressed in % of E_d_) measured in polyp surface, polyp aboral and coenosarc surface tissues of *P. damicornis* and the cell density (cells per cm^-2^). The fitting used the non-linear Levenberg Marquardt iteration algorithm and applied a power-law function (i.e., y = ax^-b^) to yield the adjusted *R^2^* and reduced χ^2^ goodness of fit parameters. Physiological and optical parameters measured for *P. damicornis* and *Favites* sp., including F_v_/F_m_, P_G_, P_N_, R_D_, L_R_, E_0_(PAR)/E_d_(PAR) were compared between healthy (day = 1) and bleached (day = 10) conditions using a two-sample Student’s *t*-test (for equal variances) or Welch’s *t*-test (for unequal variances). Statistical tests were performed in Origin Pro 9.1 (Origin, USA).

## Results

### Effect of Bleaching on Cell Density and *Symbiodinium* Pigmentation

**FIGURE 1:**
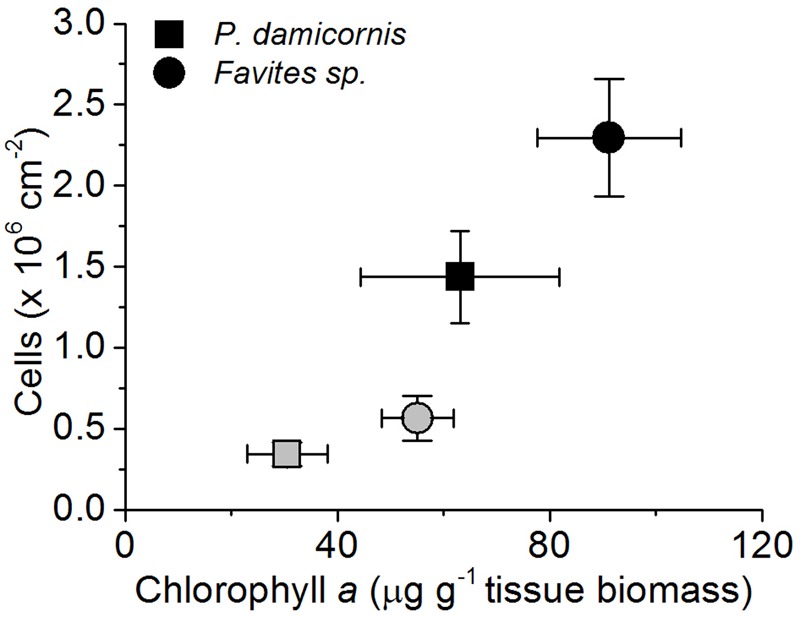
**Changes in *Symbiodinium* cell density (cells per cm^-2^ surface area) and chlorophyll *a* density (in μg chlorohyll *a* per g dry host tissue biomass) during the bleaching experiment.** Healthy corals (black symbols) and bleached corals (gray symboles) were measured at the beginning (day 1) and the end of the experiment (day 10). Data are mean ± SEM; *n* = 4–8 for cell density estimates and *n* = 6–9 for chlorophyll *a* estimates.

*Symbiodinium* cell density was significantly reduced at the end of the experiment for both *P. damicornis* and *Favites* sp. (**Figure 1**). In *P. damicornis*, the mean cell density was reduced 4.2-fold from 1.43 × 10^6^ (± SE 2.8 × 10^5^) to 3.42 × 10^5^ (± 7.5 × 10^4^ SE) cells cm^-2^ [Student’s *t*-test: *t*(10) = 3.7, *p* = 0.003; Supplementary Figure S4]. In *Favites* sp., there was a comparable fourfold reduction in cell density from 2.30 × 10^6^ (3.6 × 10^5^) to 5.6 × 10^5^ (1.4 × 10^5^ SE) cells cm^-2^ tissue surface area [Student’s *t*-test: *t*(10) = 3.3, *p* = 0.008]. Chlorophyll *a* density (per g of host tissue biomass) decreased by about 2.1- and 1.7-fold for *P. damicornis* [Student’s *t*-test: *t*(8) = 1.6, *p* = 0.146] and *Favites* sp. [Welch’s *t*-test: *t*(7.5) = 2.4, *p* = 0.047], respectively although this difference was only statistically significant for *Favites* sp. (**Figure 1**).

### Effect of Bleaching on *In vivo* Scalar Irradiance

**FIGURE 2:**
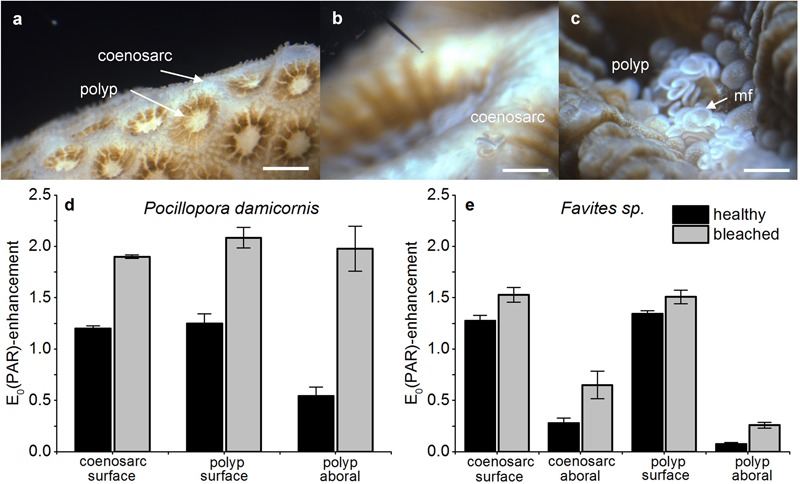
**Effect of bleaching on *in vivo* photon scalar irradiance, E_0_(PAR)-enhancement.** Typical measurement locations over the coenosarc and polyp tissues of *Pocillopora damicornis* **(a)** and *Favites* sp. **(b,c)**. Scale bar = 1 mm **(a)** and 2 mm **(b,c)**. Note the scalar irradiance microprobe **(b)** and extruded mesenterial filaments (mf) in the stressed *Favites* sp. coral **(c)**. E_0_(PAR)-enhancement was expressed as E_0_(PAR)/E_d_(PAR) and was measured in healthy and bleached *P. damicornis* **(d)** and *Favites* sp. **(e)**. Measurements were performed on the surface of coenosarc and polyp tissues as well as in the lowermost aboral tissue layer (vertical depths closest to the skeleton) for healthy (day 1) and bleached corals (day 10). Data are mean ± SE (*n* = 6 corallite-level replicates).

For all corals and measurement locations, *in vivo* photon scalar irradiance, E_0_(PAR), increased in bleached compared to healthy corals (**Figures 2a–e**). In *P. damicornis*, the greatest increase in E_0_(PAR) was measured in the aboral polyp tissues, where PAR was about 3.65-fold higher in bleached vs. healthy corals [E_0_(PAR)/E_d_(PAR) = 1.97 ± 0.21 SE and 0.54 ± 0.09 SE, respectively; Student’s *t*-test: *t*(10) = -6.1, *p* < 0.001; **Figure 2d**]. The E_0_(PAR) enhancement was less pronounced for oral polyp tissues and coenosarc oral tissues with ratios of E_0_(PAR)/E_d_(PAR) = 1.58 and 1.66, respectively (**Figure 2d**).

For *Favites* sp., E_0_(PAR) for aboral polyp tissues of bleached corals [E_0_(PAR)/E_d_(PAR) = 0.26 ± 0.03 SE] was ∼3.5 times higher than in respective healthy tissues [E_0_(PAR)/E_d_(PAR) = 0.07 ± 0.02 SE] but this difference was not significant [Welch’s *t*-test: *t*(3.1) = -2.3, *p* = 0.11]. E_0_(PAR) on the oral polyp tissue increased by 1.13 times upon bleaching [Welch’s *t*-test: *t*(3.5) = -5.5, *p* = 0.007, **Figure 2e**]. For coenosarc tissues, we similarly observed a proportionally greater E_0_(PAR) enhancement upon bleaching in aboral tissues E_0_(PAR)/E_d_(PAR) = 2.3 vs. oral tissues E_0_(PAR)/E_d_(PAR) = 1.27 (**Figure 2e**). The magnitude of E_0_(PAR) enhancement in bleached relative to healthy coral tissues was similar for *Favites* sp. and *P. damicornis*, but the absolute enhancement was different as low light niches remained in *Favites* sp. [E_0_(PAR)/E_d_(PAR) = 0.25 ± 0.03] (**Figures 2d,e**).

### Effect of Bleaching on the *In vivo* Spectral Light Environment

**FIGURE 3:**
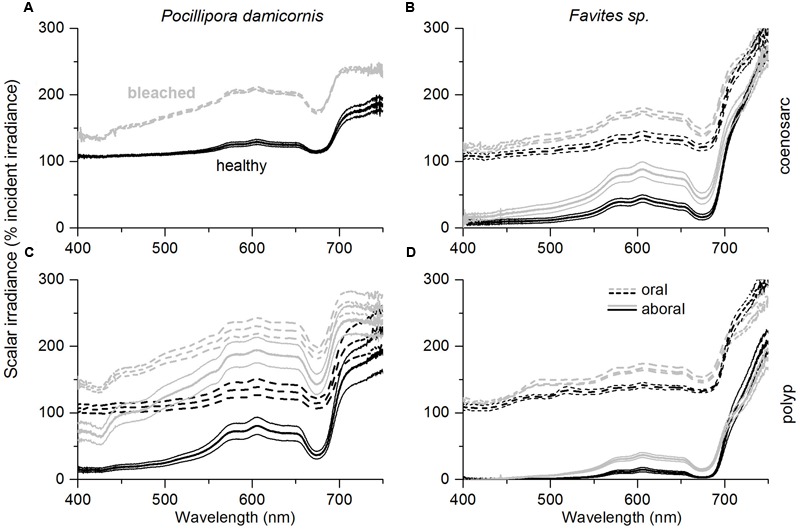
**Effect of coral bleaching on *in vivo* spectral scalar irradiance, E_0_(λ) expressed in % of the incident downwelling irradiance, E_d_(λ).** Measurements were performed on the coenosarc **(A,B)** and polyp tissue **(C,D)** of *P. damicornis* (left panel), and *Favites* sp. (right panel). Solid and dashed lines represent measurements performed in aboral tissues and at the tissue surface, respectively. Measurements were performed on healthy corals (day 1; black) and bleached corals (day 10; gray). Data are mean ± SE (*n* = 6 corallite-level replicates).

**FIGURE 4:**
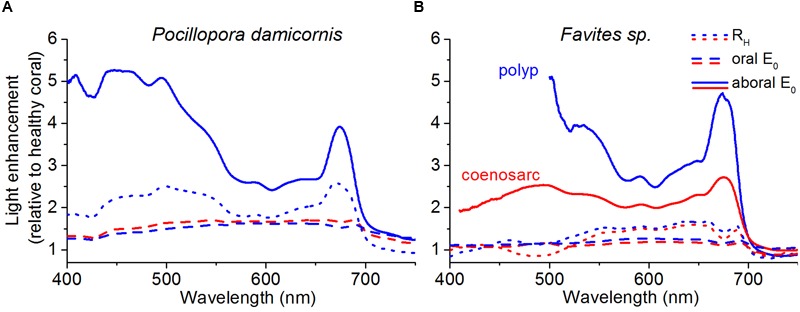
**Spectral light enhancement in bleached relative to healthy corals.** The enhancement in E_0_(λ) and *R*_H_(λ) due to bleaching was calculated as E_0_(λ)_bleached_/E_0_(λ)_healthy_ and *R*_H_(λ)_bleached_/*R*_H_(λ)_healthy_. *R*_H_(λ) (dotted lines) was measured at a distance of 1 cm from the tissue surface and E_0_(λ) was measured at the tissue surface (dashed lines) and in the aboral tissue layers closest to the skeleton (solid lines). Data was collected for both polyp (blue) and coenosarc tissues (red) for *P. damicornis* **(A)**, and *Favites* sp. **(B).** Note that *P. damicornis R*_H_(λ) measurements include areas covering both polyp and coenosarc tissues, and that E_0_(λ) measurements were not performed in aboral coenosarc tissues of *P. damicornis* due to lack of sufficient tissue thickness. Original *R*_H_(λ) spectra for bleached and healthy corals are shown in Supplementary Figure S5.

*In vivo* E_0_(λ) of bleached corals was increased relative to healthy corals (λ = 400–700 nm) for both species; however, the relative enhancement of E_0_(λ), i.e., E_0_(λ)_bleached_/E_0_(λ)_healthy_, was dependent on coral species and tissue type (**Figures 3A–D** and **4A,B**). In *P. damicornis*, E_0_(λ)_bleached_/E_0_(λ)_healthy_ in aboral polyp tissues was highest for blue light [E_0_(λ)_bleached_/E_0_(λ)_healthy_ ∼ 5 for λ = 400–500 nm] followed by far red light [E_0_(λ)_bleached_/E_0_(λ)_healthy_ ∼ 4 for λ = 670–690 nm] and least pronounced for green to red light [E_0_(λ)_bleached_/E_0_(λ)_healthy_ ∼ 3 for λ = 550–650 nm] (**Figures 3C** and **4A**). In contrast, tissue surface E_0_(λ) did not exhibit such pronounced spectral features and scalar irradiance was more evenly enhanced between 400 and 700 nm in both polyp and coenosarc tissues (**Figures 3A,C** and **4A**). A similar trend was observed in *Favites* sp., where maximal E_0_(λ) enhancement was measured in aboral polyp tissues in the blue (λ≈500 nm) and far-red (λ≈650–695 nm) (**Figures 3B,D** and **4B**). Note that no light enhancement factors were calculated for aboral polyp tissues for wavelengths less than 500 nm, due to a relatively low signal to noise ratio (a result of the almost completely depleted blue light in healthy corals). E_0_(λ) was fairly evenly enhanced between 400 and 700 nm for surface tissues of *Favites* (**Figure 4B**).

Enhancement of spectral reflectance, *R*_H_(λ)_bleached_/*R*_H_(λ)_healthy_ in *P. damicornis* followed a similar wavelength dependency as the E_0_(λ) enhancement in aboral polyp tissues, i.e., it peaked for λ = 420–550 nm and λ = 650–690 nm (**Figure 4A**; Supplementary Figure S5). However, the *R*_H_(λ)_bleached_/*R*_H_(λ)_healthy_ ratio was lower than the E_0_(λ)_bleached_/E_0_(λ)_healthy_ ratio, i.e., the increase in reflectance was lower than the increase in *in vivo* scalar irradiance of bleached tissues relative to healthy tissues (**Figure 4A**). For polyp tissues of *Favites* sp., *R*_H_(λ)_bleached_/*R*_H_(λ)_healthy_ was ∼1 and ∼1.5 for λ = 400–500 and 550–690 nm, respectively (**Figure 4B**). For coenosarc tissues, *R*_H_(λ)_bleached_/*R*_H_(λ)_healthy_ was similar to polyp tissues for wavelengths >550 nm but was <1 at 450–510 nm (**Figure 4B**). Thus reflectance measurements were not able to capture tissue depth- and tissue type-dependent dynamics in scalar irradiance, as can be seen by the more moderate increase in reflectance relative to the *in vivo scalar* irradiance during bleaching (**Figure 4**).

### *Symbiodinium* Cell Density and *In vivo* Scalar Irradiance Dynamics in *Pocillopora damicornis*

**FIGURE 5:**
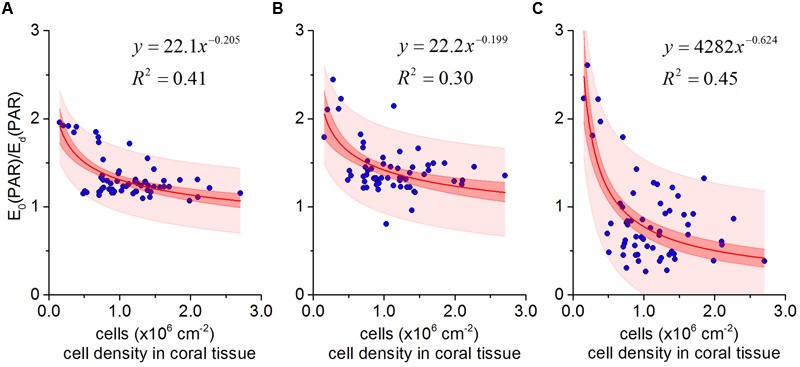
***In vivo* photon scalar irradiance enhancement E_0_(PAR)/ E_d_(PAR) as a function of *Symbiodinium* cell density in *Pocillopora damicornis* for coenosarc surface tissues **(A)**, polyp surface tissues **(B)**, and polyp aboral tissues **(C)**.** The best fit (solid red lines) in all cases was a power-law relationship (y = ax^-b^). Red and light red areas represent 95% confidence and prediction intervals, respectively.

*In vivo* E_0_(PAR) enhancement and *Symbiodinium* cell density was described by a power-law function (**Figures 5A–C**) from all data collected throughout the bleaching experiment. The best fit was obtained when *Symbiodinium* cell density was matched against E_0_(PAR) enhancement measured in aboral polyp tissues (*R*^2^ = 0.45; **Figure 5C**) (compared to *R*^2^ fits of 0.43 and 0.30 for coenosarc surface and polyp surface tissues, respectively; **Figures 5A,B**).

### Photosynthesis and Respiration of *Pocillopora damicornis*

**FIGURE 6:**
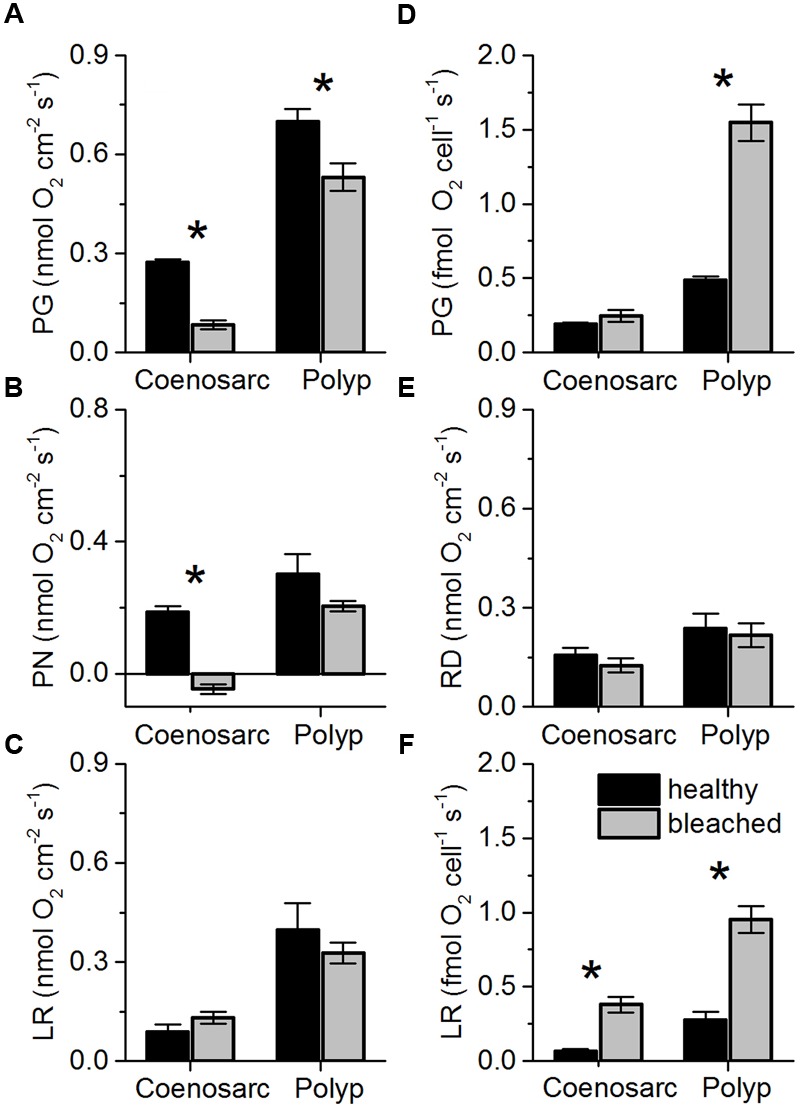
**Areal rates (in nmol O_2_ cm^-2^ s^-1^) of gross photosynthesis (A**; PG), net photosynthesis (**B**; PN), and apparent light respiration (**C**; LR = PG-PN) and dark respiration (**E**; RD) in coenosarc and polyp tissues of healthy (black bars) and bleached fragments (gray bars) of *Pocillopora damicornis*. **(D,F)** same as left panels but normalized to cell density, i.e., cell specific-rates. Data are mean ± SEM (*n* = 10–12 corallite-level replicates). Significant differences (*p* < 0.05) between bleached and healthy tissues are marked with an asterisk.

Areal rates of gross photosynthesis in bleached coenosarc and polyp tissues were reduced by 70 and 25%, respectively, when compared to the corresponding healthy tissues (**Figure 6A**), but these measurements do not account for symbiont loss after bleaching (**Figure 1**). Instead, cell-specific rates of gross photosynthesis were about 1.3- and 3.2-fold higher in coenosarc and polyp tissues, respectively, for *Symbiodinium* remaining in the bleached corals as compared to the unbleached corals (**Figure 6D**). Measurements of the maximum quantum yield of PSII showed that upon bleaching F_v_/F_m_ was reduced by about 50% as compared to healthy corals (0.38 ± 0.03 vs. 0.8 ± 0.01, respectively; mean ± SEM; Supplementary Figure S2).

Measurements of areal net photosynthesis generally showed net O_2_ production except for measurements in bleached coenosarc tissues that exhibited a net O_2_ consumption of 0.046 (± 0.015 SE) nmol O_2_ cm^-2^ s^-1^ in the light (**Figure 6B**). There was a trend toward increased holobiont light respiration in bleached vs. healthy coenosarc tissues (**Figure 6C**), while a decreased light respiration was found in polyp tissues after bleaching (0.40 ± 0.08 vs. 0.33 ± 0.03 nmol O_2_ cm^-2^ s^-1^; mean ± SEM). In contrast, apparent light respiration rates per *Symbiodinium* cell increased after bleaching by 4.2- and 2.5-fold for coenosarc and polyp tissues, respectively (**Figure 6F**). Areal dark respiration rates did not change significantly after the bleaching experiment for both coensoarc and polyp tissues (Student’s *t*-test, *p* > 0.05; **Figure 6E**).

## Discussion

Microscale light measurements revealed an average E_0_(PAR)-enhancement of ∼2 for *P. damicornis*, with maximal values of up to 2.6 within bleached polyp tissues (**Figure 5**). Such measured enhancement in *P. damicornis* can at first approximation be explained by simple light scattering events within the coral skeleton (Enriquez et al., 2005; Wangpraseurt et al., 2014a). As *Symbiodinium* cell density becomes reduced (**Figure 1**), more light penetrates toward the skeleton (**Figure 3A**) and an increased fraction of this light is backscattered (Enriquez et al., 2005). For a flat isotropically scattering skeleton with a reflectance, *R*, the average path length of upwelling photons is twice that of photons in the incident collimated light beam traversing a thin layer of coral tissue (ignoring tissue scattering) (Kühl and Jørgensen, 1994). Here, the contribution of the coral skeleton reflectance on the scalar irradiance can be calculated as (Kortüm, 2012): E_o_ = (1 + 2R)E_d_, where *E*_d_ is the incident downwelling irradiance. The flux absorbed by *Symbiodinium* can thus be estimated as Φ_abs_ = 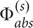 + 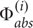 = (1 + 2R)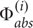, where 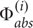 is the absorption of the incident beam, i.e., 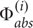 = A × E_d_, where *A* is the absorption cross-section of *Symbiodinium* (Enriquez et al., 2005). Diffuse reflectance of *P. damicornis* skeletons in our study was on average *R* = 0.5 (data not shown), which is comparable to previous measurements (*R* = 0.36, Swain et al., 2016). As such, our theoretical calculations would predict an E_0_(PAR)-enhancement of 2, a value consistent with our direct measurements for the thin bleached coenosarc tissues of *P. damicornis* (**Figure 5A**).

In contrast to the consistent theoretical and measured E_0_(PAR)-enhancement values for *P. damicornis* coenosarc tissues, we observed an E_0_(PAR) enhancement of >2 for polyp tissues (**Figures 5B,C**). E_0_(PAR) enhancement of >2 suggests an additional scattering contribution from the coral tissue (Wangpraseurt et al., 2014a) and/or from the concave corallite architecture (Ow and Todd, 2010). Indeed, measurements for bare skeletons (Supplementary Figure S1) revealed a higher E_0_ within the corallite than over the coenosteum, suggesting that the small concave corallite for *P. damicornis* (width and length ∼1 mm; Nothdurft and Webb, 2007) effectively homogenizes and redirects the incident radiation, through multiple reflections from the skeletal walls toward the center of the corallite, whereas light escapes more easily as diffuse reflectance from the rather flat coenosteum. Given the thin tissue in *P. damicornis*, multiple scattering by such millimeter-sized skeletal structures likely contributes to the observed higher E_0_(PAR)-enhancement of *Symbiodinium* in polyp vs. coenosarc tissues in the intact living coral (**Figure 4A**). However, the E_0_(PAR)-enhancement observed in intact bleached *P. damicornis* was still higher than for bare skeletons indicating additional light enhancement through tissue scattering. It is possible that diffusely backscattered photons from the skeleton are trapped by the tissue (see detailed discussion in Wangpraseurt et al., 2014a) and/or that the scattering coefficient is higher for coral tissue than for coral skeleton, as is the case for *Favites* sp. (Wangpraseurt et al., 2016a).

The optical feedback hypothesis predicts an inverse power-law relationship between *Symbiodinium* cell density and *in vivo* light exposure, i.e., the rate of light field ‘amplification’ increases as symbiont cell density decreases (Teran et al., 2010; Swain et al., 2016). For a small cup-shaped polyp, with low tissue absorption and scattering, the greatest effect of corallite architecture on light scattering is expected in close proximity to the skeletal surface at the center of the corallite (Teran et al., 2010; Wangpraseurt et al., 2016a). Our measurements for *P. damicornis* support these predictions since the highest rate of light enhancement was measured within aboral polyp tissues (*R*^2^ = 0.45, **Figure 5C**). Scattering by (sub)millimeter-sized skeletal surface elements in such a thin-tissued coral with small polyps thus play a key role in the measured light enhancement dynamics upon bleaching.

Coral bleaching also involves a change in the spectral exposure of the remaining symbionts (**Figure 4**). In a normally pigmented healthy coral, blue light (400–450 nm) is rapidly attenuated vertically within the coral tissue due to peak light absorption by Chl *a*, leaving symbionts in aboral tissue layers exposed to a green to red shifted light spectrum (**Figure 3**; Szabó et al., 2014). As coral bleaching progresses, symbionts in aboral tissues are progressively exposed to greater amounts of blue light (**Figure 4**). It is important to note that such spectral changes could not be captured solely based on surface scalar irradiance or diffuse reflectance measurements (**Figure 4**), highlighting the relevance of depth resolved measurement of *in vivo* scalar irradiance. Minor changes in the spectral composition of light can affect *Symbiodinium* photosynthesis *in vivo* (Wangpraseurt et al., 2014c) and it will be interesting in the future to study the effect of changing spectral exposure on symbiont photosynthesis along a vertical gradient within the tissue (Lichtenberg et al., 2016).

Our data demonstrated the presence of species-specific patterns of light modulation, whereby light gradients were alleviated in polyp tissues of *P. damicornis* but remained present in polyp tissues of *Favites* sp. after bleaching (**Figures 3A–D**). *Favites* sp. are characterized by thick, light scattering tissues (Wangpraseurt et al., 2014a, 2016a) with host pigments such as GFP (Salih et al., 2000; Lyndby et al., 2016) and mycosporine-like amino acids (Dunlap and Shick, 1998; Lesser and Farrell, 2004) that often remain present during bleaching (Smith et al., 2013). Although, *P. damicornis* does also have a GFP-like pigment (Takabayashi and Hoegh-Guldberg, 1995), the GFP-like pigments in *Favites* sp. are arranged in a chromatophore system that strongly enhances light scattering (Lyndby et al., 2016; Wangpraseurt et al., 2016b) facilitating a steep light attenuation along the enhanced optical path within thick scattering coral tissue (Wangpraseurt et al., 2012; Lyndby et al., 2016). Our finding of low light levels in the aboral polyp tissues therefore suggests that tissue background scattering and absorption effectively attenuate light even in bleached tissues of *Favites* sp. Additionally, differences between *P. damicornis* and *Favites* sp. in skeleton optical properties are likely (Marcelino et al., 2013; Swain et al., 2016; Wangpraseurt et al., 2016a), which would further affect the fraction of light ‘amplification’ by the skeleton (Marcelino et al., 2013). Thus our results indicate that symbionts in *P. damicornis* will be more severely affected by ‘optical feedback’ than symbionts in *Favites* sp. during coral bleaching. The moderate light environment in aboral polyp tissues of bleached *Favites* sp. could facilitate photoprotection of remaining cryptic *Symbiodinium* sp. (Silverstein et al., 2012), which could be a key determinant for symbiont repopulation and subsequent coral recovery from bleaching (Rowan et al., 1997).

However, it is likely that a variety of structural design solutions exist that provide photoprotection on different spatial scales (see also Yost et al., 2013). For a branching coral, such as *P. damicornis*, light attenuates along the branch due to shading by neighboring branches (Kaniewska et al., 2011). The magnitude of light attenuation along a single branch is similar to the light attenuation observed within the polyp tissues of *Favites* sp. (compare Kaniewska et al., 2011 and Wangpraseurt et al., 2012). Thus the presence of moderate light microenvironments is expected for *P. damicornis* due to its colony-level architectural complexity, which was however, not assessed in the present study. We visually observed that corals bleached first over the coenosarc tissue area, while symbionts remained within the tentacles of *P. damicornis* despite the enhanced *in vivo* light environment in polyp tissues. It was not possible to measure the light microenvironment within the highly contractile tentacle tissue and it remains unknown, whether a more moderate light microenvironment remained within the tentacles facilitating resistance to bleaching. Additionally, it is possible that bleaching was alleviated for tentacle tissues because of tentacle movement leading to enhanced gas exchange (Shashar et al., 1993) and possibly reducing the build-up of high O_2_ levels alleviating oxidative stress.

We observed increased symbiont cell-specific rates of gross O_2_ evolution and light respiration in bleached relative to healthy *P. damicornis*, although F_v_/F_m_ values and areal photosynthetic rates (gross and net) decreased (**Figure 6**). Numerous studies have reported lowered F_v_/F_m_ values (Warner et al., 1999; Jones et al., 2000; Supplementary Figure S2) and reduced net photosynthesis of *Symbiodinium* in response to thermal and/or light stress (Iglesias-Prieto and Trench, 1994; Warner et al., 1996, 1999), but few studies have measured gross O_2_ evolution independent of light respiration (Kühl et al., 1995; Abrego et al., 2008; Schrameyer et al., 2014). A lowering of F_v_/F_m_ is often interpreted as a sign of photoinhibition of *Symbiodinium* and can result from the formation of non-functional PS II reaction centers, i.e., a downregulation of PSII efficiency that still allows for O_2_ evolution to occur (Hill et al., 2004). The high cell-specific gross photosynthetic rate (**Figure 6D**) could be explained as a downregulation of PSII efficiency counteracting the rapidly increasing *in vivo* irradiance (**Figure 4**). It is also possible that the observed dynamics reflect photoacclimation of *Symbiodinium*, as high light acclimation involves an increase in the functional absorption cross section of PSII that leads to a lowering of F_v_/F_m_ (Hennige et al., 2008; Suggett et al., 2009). Additionally, it is possible that bleaching affected the symbiont community, potentially favoring more efficient photosynthesisers under high temperature (Roth, 2014; Sampayo et al., 2016).

The relative increase of cell-specific photosynthesis for bleached relative to healthy corals will depend on the quantity of incident solar radiation. Assuming a scenario where incident radiation saturates photosynthesis in all algal cell layers of a healthy coral, the *in vivo* light enhancement due to bleaching would not allow for a further enhancement of photosynthesis. Likewise, a faster increase in temperature would lead to more severe photoinhibtion and ultimately reduced photosynthetic rates. Additionally, our data showed that cell density (per surface area) declined to a greater extent than chlorophyll content (per g host tissue biomass). The reason for this mismatch remains unknown but could be related to reduced tissue thickness, which would affect chlorophyll but not cell density data, and/or potentially reflect the limited capacity of the HPLC to detect the Chl *a* signals in our microsamples. Normalization of gross photosynthesis data per chlorophyll *a* content of the corals showed that gross photosynthesis was only enhanced for polyp tissues (by about 1.6-fold) of bleached vs. healthy *P. damicornis* corals (data not shown). The enhancement of cell-specific photosynthesis during coral bleaching should thus be interpreted with caution and needs further investigation.

Light respiration significantly increased during the bleaching experiment, accounting for about 35% of gross photosynthesis in healthy tissues up to about 150% of gross photosynthesis in bleached tissues (**Figure 6**). Enhanced, apparently light-driven, O_2_ consumption is a sign of increased electron flow through alternative electron pathways, of which the Mehler reaction is especially prevalent in *Symbiodinium* (Roberty et al., 2014). While an upregulation of the Mehler reaction has been interpreted as a photoprotective mechanism to decrease excitation pressure on PSII under high light (Roberty et al., 2014), the Mehler reaction generates ROS that can readily lead to oxidative stress, if antioxidants such as superoxide dismutase and ascorbate peroxidase are not upregulated as well (Krueger et al., 2014; Roberty et al., 2014). Together, the enhanced metabolic activity likely reflects a response of *Symbiodinium* to the strongly accelerated *in vivo* light environment (**Figure 5**) showing that coral microscale optics exert a key role in coral photophysiology.

Climate change-related coral bleaching is arguably the prime cause of global reef decline and better predictions of coral bleaching susceptibility rest on understanding the mechanisms at play (Hoegh-Guldberg, 1999; Hoegh-Guldberg et al., 2007; Weis, 2008). The optical feedback hypothesis (Enriquez et al., 2005), whereby *Symbiodinium* undergoes light exposure ‘amplification’ during coral bleaching (Enriquez et al., 2005; Swain et al., 2016) has lacked experimental evidence in terms of direct measures of the tissue light field before and during coral bleaching. Here, we give the first experimental proof that the *in vivo* scalar irradiance in corals is enhanced during coral bleaching due to changes in the balance between absorption and scattering upon loss of *Symbiodinium*. Light amplification differs between coral hosts, as tissue light gradients and optical shelter remained in the massive coral *Favites* sp. but were alleviated in *P. damicornis*. The finding of optical shelter in bleached *Favites* sp. tissues implies a photoprotective microenvironment that could sustain coral resilience by facilitating the repopulation from cryptic *Symbiodinium* after stress. Our study shows that coral bleaching can in fact enhance cell-specific photosynthetic rates of remaining *Symbodinium*, which is interpreted as a response to the accelerated *in vivo* light exposure during bleaching. We conclude that coral microscale optics have a fundamental role in shaping the radiative exposure of *Symbiodinium* and thus photophysiological stress responses during coral bleaching.

## Author Contributions

All authors designed the experiment. JH and DW analyzed the data. DW, MK, DS, and JH interpreted the data. DW performed microsensor measurements and JH performed symbiont cell density and tissue surface area estimates. DW, JH, AL performed the experimental bleaching treatment. MK and PR provided new reagents, materials and tools: DW, MK, and JH wrote the paper with editorial input and consent from all co-authors.

## Conflict of Interest Statement

The authors declare that the research was conducted in the absence of any commercial or financial relationships that could be construed as a potential conflict of interest.

## Abbreviations

E_d_(PAR): incident downwelling photon irradiance (μmol photons m^-2^ s^-1^)
E_0_(PAR): photon scalar irradiance (μmol photons m^-2^ s^-1^)
E_0_(λ): spectral scalar irradiance (μmol photons m^-2^ s^-1^ nm^-1^)
E_0_(PAR)_bleached_/E_0_(PAR)_healthy_: photon scalar irradiance enhancement of a bleached coral relative to a healthy coral
E_0_(λ)_bleached_/E_0_(λ)_healthy_: spectral scalar irradiance enhancement of a bleached coral relative to a healthy coral
L_R_: light respiration
NIR: near-infrared radiation
PAR: photosynthetically active radiation (400–700 nm)
P_N_: net photosynthesis
P_G_: gross photosynthesis
R_D_: dark respiration
R_H_(λ): spectral holobiont reflectance
R_H_(λ)_bleached_/R_H_(λ)_healthy_: holobiont reflectance enhancement of a bleached coral relative to a healthy coral
