# Supplementary material for: In vivo Microscale Measurements of Light and Photosynthesis during Coral Bleaching: Evidence for the Optical Feedback Loop?
Source: Front Microbiol. 2017 Jan 24;8:59. doi: 10.3389/fmicb.2017.00059 (PMC5258690; doi:10.3389/fmicb.2017.00059)
Supplement: Supplementary file 1 [file Data_Sheet_1.DOCX]

**Supporting information**

*In vivo* microscale measurements of light and photosynthesis during coral bleaching: evidence for the optical feedback loop?

**Daniel Wangpraseurt^1,2^*, Jacob B. Holm^1^, Anthony W. D. Larkum^2^, Mathieu Pernice^2^, Peter J. Ralph^2^, David J. Suggett^2^, Michael Kühl^1,2^***

^1^Marine Biological Section, Department of Biology, University of Copenhagen, Helsingør, Denmark

^2^Climate Change Cluster, Department of Environmental Sciences, University of Sydney, Sydney, NSW, Australia.

*** Correspondence:** [daniel.wangpraseurt@bio.ku.dk](mailto:daniel.wangpraseurt@bio.ku.dk), [mkuhl@bio.ku.dk](mailto:mkuhl@bio.ku.dk)

**1. Supplementary methods**

**1.1 Combined irradiance and thermal stress treatment for *Favites sp*.**

Symbiont loss of *Favites sp.* corals was accelerated with a combined irradiance and thermal stress treatment. Colonies were removed from the main experimental tank during three subsequent evenings (days 7-9) and kept in a simple indoor aquarium set-up for about 7 hours (from around 5 pm to midnight). The indoor aquarium (about 40 x 40 x 20 cm) setup provided the same water temperature as the outdoor set-up but allowed for extending the illumination period and delivering excess irradiance. *Favites* colonies were illuminated with a photon irradiance of E_d_(PAR)= 2500 μmol photons m^-2^ s^-1^ as delivered by a fiber-optic tungsten halogen lamp (Schott KL-2500, Germany). During incubation, aerated water was provided at an intermediate flow rate of about 2-3 cm s^-1^.

**1.2 Scalar irradiance measurements on intact corals**

The scalar irradiance microsensors were mounted on a motorised micromanipulator (Pyro-Science GmbH, Germany) and oriented at a 45° angle relative to the vertically incident light (E_d_(PAR) = 400 μmol photons m^-2^ s^-1^) provided by a fiber-optic tungsten halogen-lamp equipped with a collimating lens (Schott KL-2500, Germany). The probes were connected to a fiber-optic spectrometer (USB2000+, Ocean Optics, USA) interfaced to a PC running the manufacturer’s software (Spectrasuite, Ocean Optics, USA). Depth profiles of scalar irradiance were performed by positioning the sensor at known distances from the coral tissue surface (defined as depth=0 µm) by means of the micromanipulator and observed under a stereo-microscope (Olympus SZX7, Tokyo, Japan).

**1.3 Reflectance and scalar irradiance measurements of bare skeletons**

Prior to reflectance and scalar irradiance measurements on *Pocillopora damicornis* and *Favites* sp. skeletons, the coral tissue was removed with an airgun, thereafter the skeletons were carefully cleaned off any remaining debris. The reflectance and scalar irradiance measurements were performed on submersed skeletons using the same procedures as on intact corals. Residual air trapped between skeleton ridges was released by carefully tapping the skeleton and brushing its surface underwater with a paintbrush. Scalar irradiance measurements on skeletons were performed on similar measurement areas as on the intact corals, i.e., on the coenosteum and within the centre of the corallite (Fig. S1).

**1.4 Variable chlorophyll fluorescence imaging**

Variable chlorophyll *a* fluorescence imaging was performed using a commercial imaging pulse amplitude modulated chlorophyll *a* fluorometer (Maxi-Version, I-PAM, Heinz Walz GmbH, Germany). For each measurement, a single coral fragment was placed in a heated and aerated water bath, where it was dark adapted for about 20 minutes before a saturating pulse (2500-3000 µmol photons m^-2^ s^-1^ for 0.8 s) was applied to determine the maximum quantum yield of PSII, F_v_/F_m_=(F_o_-F_m_)/F_m_. For each fragment, three randomly selected branch tips were monitored using the IMAGING-PAM software (Imaging Win v.2, Heinz Walz GmbH, Germany) for each of 3 fragments *of Pocillopora damicornis* per measurement day. F_v_/F_m_ data was averaged for measurements performed under healthy (day=1) and bleached (day=10) conditions (Fig. S2).

**1.5 Zooxanthellae density**

Zooxanthellae density in *Favites* sp. colonies was determined using the microsampling technique (Kemp et al., 2008). Coral tissue was collected through careful insertion and rotation of an 18 gauge needle into the coral polyp or coenosarc tissue (Fig. S3). The tissue biomass was collected with a 3 mL syringe, expelled to 2 mL microcentrifuge tube and centrifuged at 4500 *g* for 5 min to separate algal cells from host tissue (Middlebrook et al., 2010). The supernatant was removed and the algal pellet was resuspendend in sterilized Milli-Q water. Zooxanthellae density was measured using a Sedgewick (ProSciTech s8050, Kirwin, Queensland, Australia) rafter cell. Ten 1 µL cells were counted within a 1 mL slide and averaged per sample. The surface area from which tissue was collected was photographed (Olympus DP71-microscope camera) and subsequently analysed using the open software Image J (v 1.47, National Institute of Health, Bethesda, MD, USA). This involved calibrating the pixel into actual units (mm) and then outlining the sampled *Favites* sp. tissue area via a manual drawing tool. Image J then allows to easily quantify the encircled surface area (using the function analyze > measure) of the calibrated image.

For *P. damicornis*, the uppermost part (maximum of 0.5 to 0.8 cm in length) of the branch tip of interest, i.e., the same area as used for microsensor measurements was collected using a bone cutter. The radius and height of the collected tip were measured to the nearest mm using a calliper (Fig. S3c). Surface area *A* (cm^-2^) was calculated as $A=2\pi rh+\pi r^{2}$, where *h* and *r* are the height and radius of the branch tip, respectively (Naumann et al., 2009). Note that this cylinder only includes the sides and the top of the branch, as the base is not covered by living tissue. The branch tip was crushed using an agate mortar and pestle (Sigma Aldrich, USA) and the entire coral tissue slurry including skeletal material was transferred with a known amount of sterilized milli-Q to a microcentifuge tube. Centrifugation and cell counts were then performed as described above.

**1.6 High performance liquid ion chromatography (HPLC)**

Freeze dried samples were homogenised and extracted in acetone (400 µL x 2, 200 µL x 1) using an ultrasonic probe under dark and cold conditions. Combined extracts were then filtered through a 13mm syringe filter (0.2 μm PTFE) and stored in amber coloured HPLC glass vials at -20°C freezer. An Agilent 1290 HPLC system equipped with a binary pump with integrated vacuum degasser, thermostatted column compartment modules, an Infinity 1290 autosampler, and a PDA detector was used for the analysis. Column separation was performed using an Agilent's Zorbax Eclipse XDB C8 HPLC (4.6 mm × 150 mm) and guard column using a gradient of TBAA: MeOH (30:70) (solvent A) and Methanol (Solvent B) as follows: 0–22 min, from 5 to 95% B; 22–29 min, 95% B; 29-31 min, 5%B; 31-40 min, column equilibration with 5%B. A sandwich injection approach was set using the auto injector program, where the TBAA buffer and samples were drawn alternatively in the sequence, 310: 30: 300: 30: 230 (µL) and then mixed in the loop and injected. A complete pigment spectrum from 270 to 700 nm was recorded using the PDA detector with 3.4 nm bandwidth. Calibration was performed using individual pigment standards (DHI, Denmark). Chl *a* content was normalised to the freeze dried tissue biomass.

**2. Supplementary figures**

**
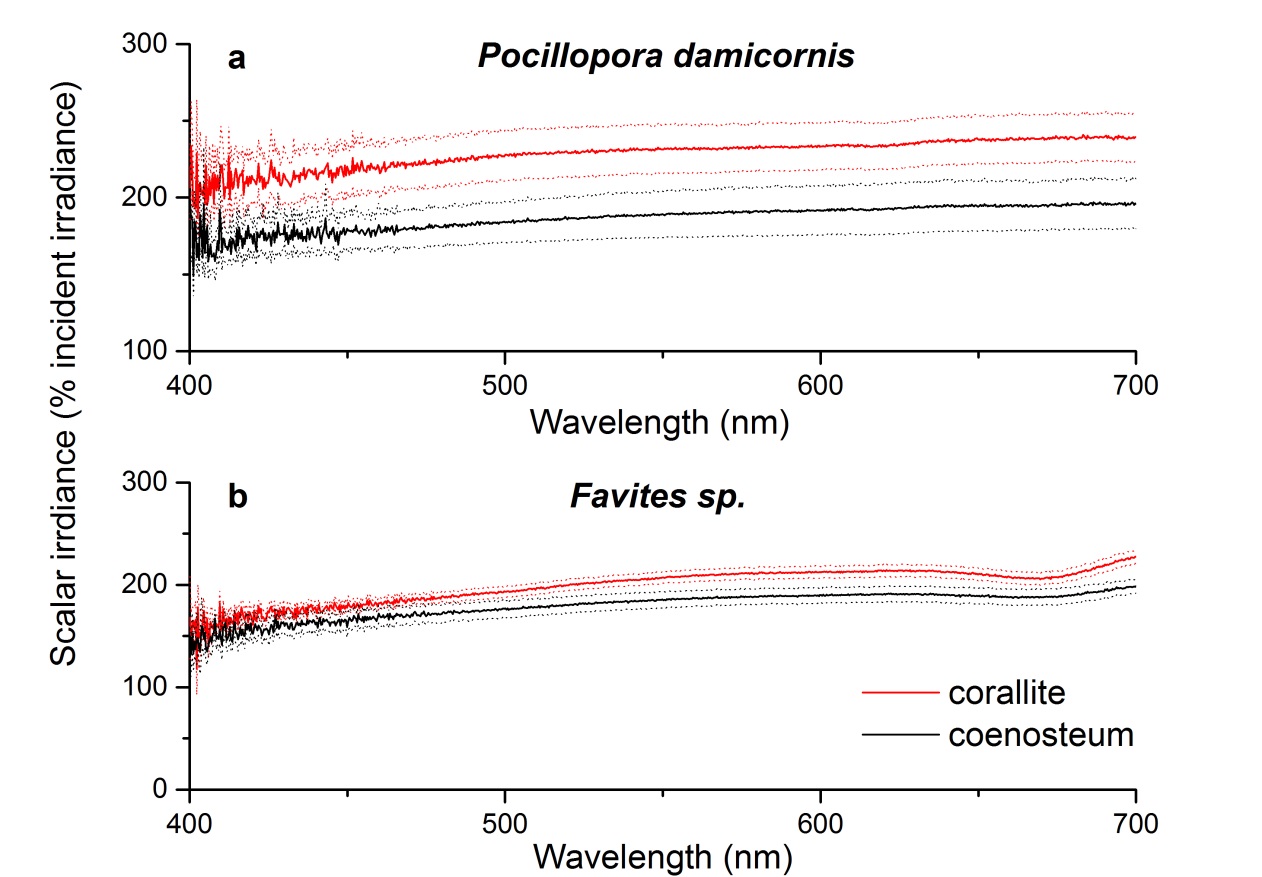
**

**Fig S1. Spectral scalar irradiance measurements on bare coral skeletons.** Spectral scalar irradiance (in % of incident irradiance) was measured on 3 corallite (red lines) and coenosteum (black lines) areas for each of 3 bare skeleton fragments of *Pocillopora damicornis* (**a**) and *Favites sp.* (**b**). Solid lines are means and dotted lines are standard errors (*n*=9).

**
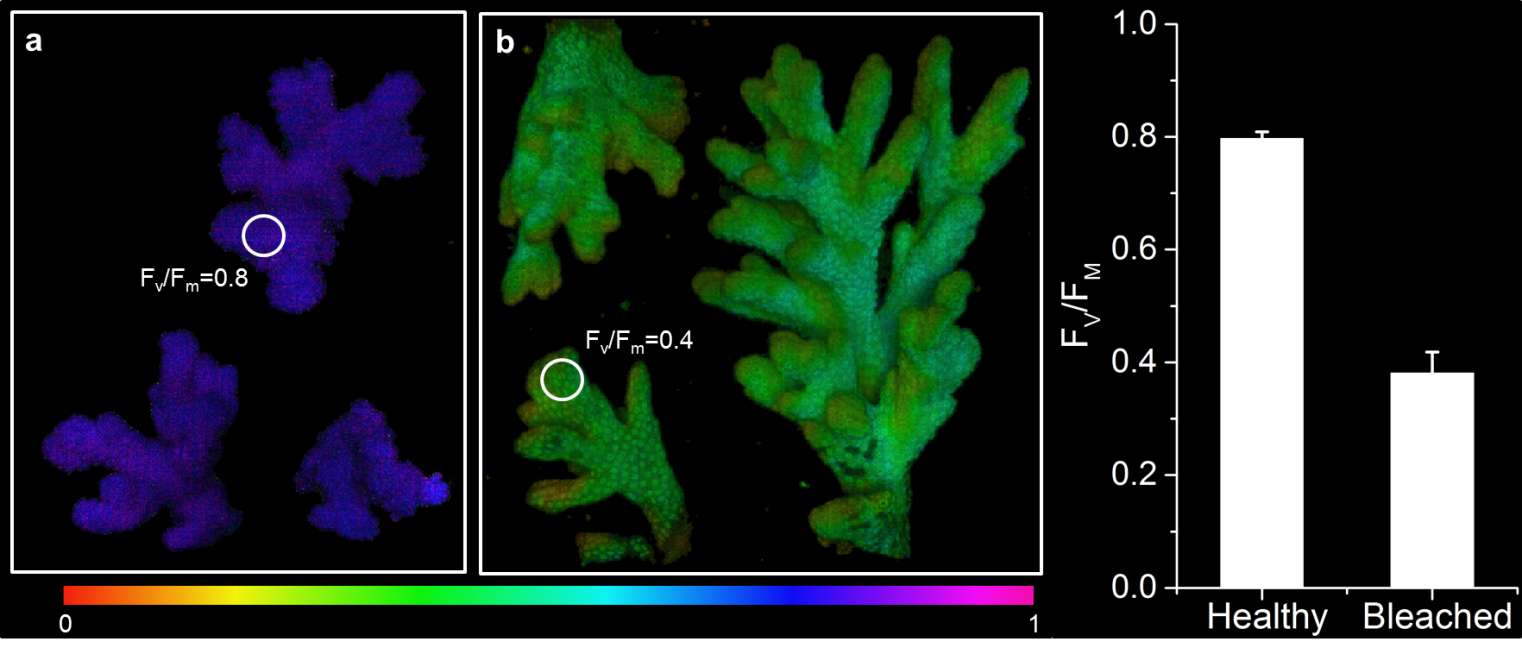
**

**Figure S2. Variable chlorophyll fluorescence imaging of corals.** Example images of the maximum quantum yield of PSII (F_v_/F_m_) measured in healthy (**a**) and bleached (**b**) *Pocillopora damicornis* corals. Average F_v_/F_m_ values (±SE, *n*=9) for 3 areas of interest (example area shown as white circle) from each of 3 fragments (**c**).


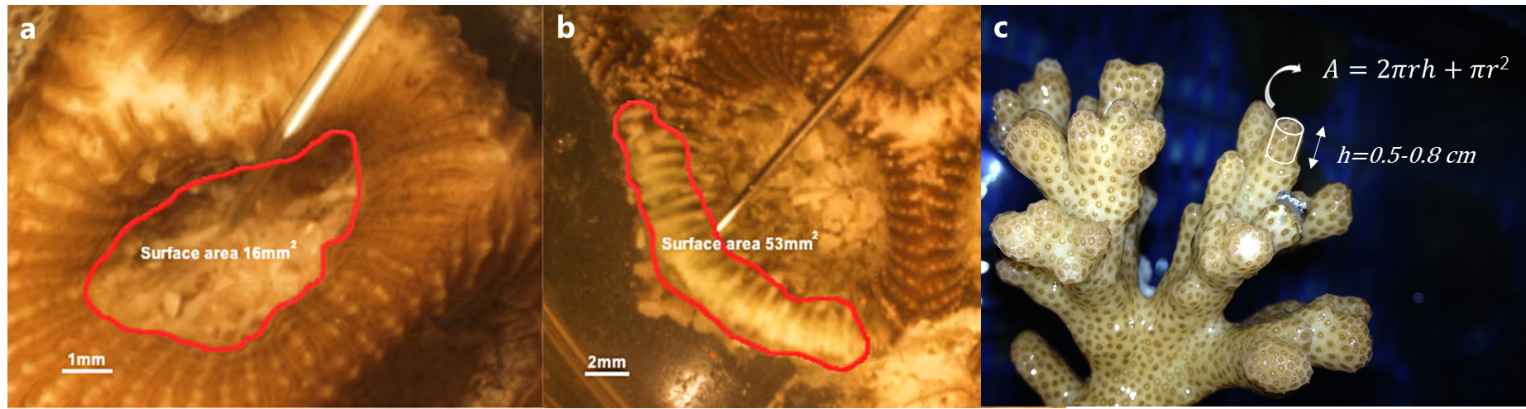


**Figure S3. Millimetre scale determination of coral tissue surface area.** The sampled *Favites* sp. tissue area was outlined in Image J (indicated as red line) for polyp areas **(a)** and coenosarc areas **(b)**. The uppermost part of the branch tip (with a maximum height of 0.5-0.8 cm) of *Pocillopora damicornis* was collected and the surface area *A* calculated assuming cylinder geometry **(c)**. The needle was used as scaling reference.

**
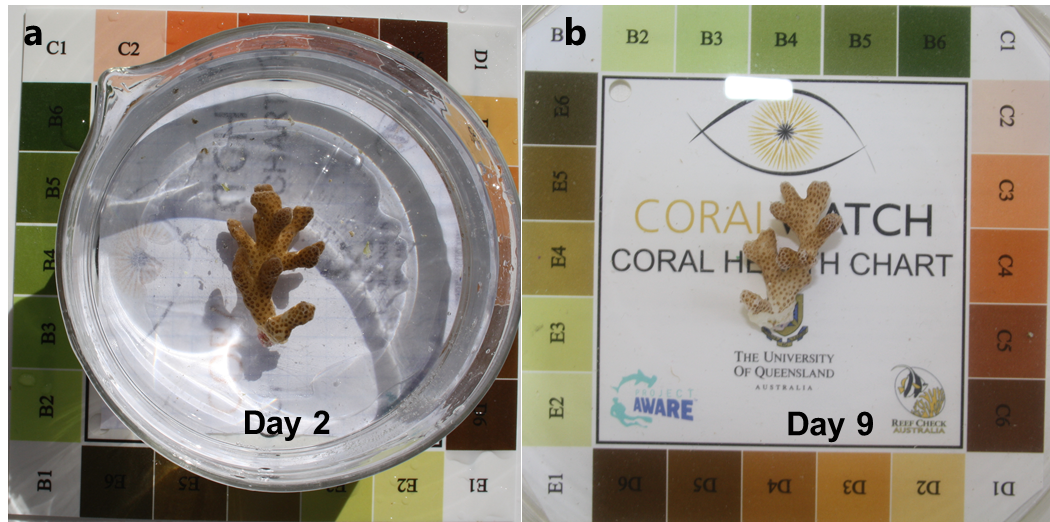
**

**Figure S4. Visual observation of symbiont loss during thermal stress.** Photograph of an example fragment of ***Pocillopora damicornis*** on day 2 (**a**) and the same fragment on day 9 (**b**).





**Figure S5. Effects of coral bleaching on coral spectral reflectance *R_H_*.** Measurements were performed on healthy (black lines) and bleached tissues (red lines) for *P.damicornis* (which included both coenosarc and polyp tissue areas) (a), *Favites sp.* coenosarc tissue (b), and *Favites sp*. polyp tissues (c). Data are means ± SEM (n=9).

**3. Supplementary references:**

Kemp, D., Fitt, W., and Schmidt, G. (2008). A microsampling method for genotyping coral symbionts. *Coral Reefs* 27**,** 289-293.

Middlebrook, R., Anthony, K.R., Hoegh-Guldberg, O., and Dove, S. (2010). Heating rate and symbiont productivity are key factors determining thermal stress in the reef-building coral *Acropora formosa*. *J Exp Biol* 213**,** 1026-1034.

Naumann, M.S., Niggl, W., Laforsch, C., Glaser, C., and Wild, C. (2009). Coral surface area quantification–evaluation of established techniques by comparison with computer tomography. *Coral Reefs* 28**,** 109-117.
